# Feasible outcome of blinatumomab followed by allogeneic hematopoietic cell transplantation for adults with Philadelphia chromosome‐negative acute lymphoblastic leukemia in first salvage

**DOI:** 10.1002/cam4.2680

**Published:** 2019-11-05

**Authors:** Jae‐Ho Yoon, Gi June Min, Sung‐Soo Park, Silvia Park, Sung‐Eun Lee, Byung‐Sik Cho, Ki‐Seong Eom, Yoo‐Jin Kim, Hee‐Je Kim, Chang‐Ki Min, Seok‐Goo Cho, Dong‐Wook Kim, Jong Wook Lee, Seok Lee

**Affiliations:** ^1^ Department of Hematology Catholic Hematology Hospital and Leukemia Research Institute Seoul St. Mary's Hospital College of Medicine The Catholic University of Korea Seoul Korea

**Keywords:** acute lymphoblastic leukemia, allogeneic, blinatumomab, first salvage, hematopoietic cell transplantation

## Abstract

In adult patients with relapsed or refractory (R/R) Philadelphia chromosome‐negative (Ph‐negative) B‐cell presursor acute lymphoblastic leukemia (BCP‐ALL), complete remission (CR) and overall survival (OS) rates are poor. We analyzed treatment outcomes and prognostic factors for 32 adult patients with R/R Ph‐negative BCP‐ALL who received blinatumomab at first salvage. Patients who achieved CR proceeded to allogeneic hematopoietic cell transplantation (allo‐HCT). At the time of blinatumomab treatment, 11 patients (34.3%) were primary refractory, 10 (31.4%) had relapsed with first CR duration (CRD1) ≥12 months, and 11 (34.3%) had relapsed with CRD1 <12 months. After the first blinatumomab cycle, 22 (68.8%) achieved CR. At the end of the second cycle, 20 of the 22 patients remained in persistent CR, and 1 patient achieved new CR. The overall minimal residual disease negativity rate was 75% among evaluable patients with persistent CR. Patients with CRD1 <12 months were associated with poorer response to blinatumomab. Twenty (62.5%) of 32 patients underwent allo‐HCT in blinatumomab‐induced CR. After a median follow‐up of 15.2 months, the 1‐year OS rates for all patients and patients receiving allo‐HCT in CR were 55.5% (median OS, 18.2 months) and 70.7%, respectively. Patients with CRD1 <12 months, extramedullary disease (EMD), and high peripheral blood blasts were associated with poorer OS. Blinatumomab is effective for achieving good quality CR and bridging to allo‐HCT for adult patients with R/R Ph‐negative BCP‐ALL in first salvage. The role of blinatumomab in patients with CRD1 <12 months, EMD, or high tumor burden should be evaluated in future trials.

## Graphical abstract

Blinatumomab is effective for achieving good quality CR and bridging to allo‐HCT for adult patients with R/R Ph‐negative BCP‐ALL in first salvage. The role of blinatumomab in relapsed patients with short CR duration, extramedullary disease, and high tumor burden should be cautiously considered due to poor response.

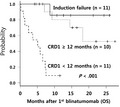

## 1 INTRODUCTION

In adult patients with B‐cell presursor acute lymphoblastic leukemia (BCP‐ALL), complete remission (CR) rates are 90% and long‐term overall survival (OS) is 30%‐60% after intensive chemotherapy with or without allogeneic hematopoietic cell transplantation (allo‐HCT).^1^, ^2^, ^3^ However, many patients with BCP‐ALL relapse and die from leukemia progression. Relapse remains an unmet clinical challenge for physicians even after allo‐HCT, which is the gold standard procedure for adult patients in first CR.

After relapse, CR rates are estimated to be much lower with the use of standard salvage chemotherapy, and survival outcomes are very poor.^4^, ^5^, ^6^ For safe salvage and bridge therapy to allo‐HCT, several immune‐based treatments such as bi‐specific T‐cell engager (BiTE),^7^, ^8^ chimeric antigen receptor engineered T‐cell therapy (CAR‐T),^9^ and antibody‐drug conjugate platform have been attempted.^10^, ^11^, ^12^ More recently, the combination of immunotherapy with lower intensity cytotoxic therapy has been developed and provides very promising outcomes for the treatment of ALL.^13^, ^14^, ^15^, ^16^

Blinatumomab was the first BiTE antibody that demonstrated both a good safety profile and relevant antileukemic activity.^17^, ^18^, ^19^, ^20^, ^21^ Blinatumomab contains binding regions for the B‐cell lineage specific CD19 and the invariant CD3ε subunit of the T‐cell receptor present on T lymphocytes, which is readily produced in high amounts with reliable purification and stability.^22^ The most rapid killing is mediated by perforin‐granzyme activity of CD8^+^ T‐cells, but CD4^+^ T‐cells are also stimulated by blinatumomab.^23^ This novel drug is a powerful therapeutic option in patients with relapsed/refractory (R/R) BCP‐ALL in major clinical trials, especially for patients treated earlier or those with minimal disease burden.^17^, ^18^, ^19^, ^20^ However, remission rates, survival outcomes, and complications of this agent in real‐world practice are not widely reported. The clinical effects of blinatumomab followed by allo‐HCT as a first salvage treatment have yet to be elucidated, and the prognostic factors for blinatumomab response and survival outcome should be analyzed.

This study analyzes the treatment outcomes and prognostic factors for adult patients with R/R Philadelphia chromosome‐negative (Ph‐negative) BCP‐ALL who received blinatumomab in first salvage. Patients who achieved CR proceeded to allo‐HCT as early as possible if a donor was available.

## 2 PATIENTS AND METHODS

### 2.1 Enrolled patients

During the period between November 2016 and November 2018, 32 consecutive patients with R/R Ph‐negative BCP‐ALL (median age, 44 years [range, 18‐70 years]) were treated with blinatumomab as a first salvage treatment. According to the Korean national health insurance guidelines, patients with Ph‐negative BCP‐ALL who failed to achieve hematologic CR after first‐line induction chemotherapy were treated with blinatumomab. In addition, relapsed patients after consolidation chemotherapy or previous allo‐HCT were also priority candidates for blinatumomab salvage treatment. The Institutional Review Board of The Catholic University of Korea approved this study, and written informed consent was obtained from all patients (KC19RESI0125). This study was conducted in accordance with the Declaration of Helsinki.

### 2.2 Treatment strategy

To reduce leukemia burden and cytokine release syndrome (CRS), all patients received prephase treatment with dexamethasone (10 mg intravenously every 12 hours for 4 days, 80 mg in total). During the first cycle, blinatumomab was continuously infused for 4 weeks (9 μg/d for the first 7 days and 28 μg/d thereafter). After a 2‐week treatment‐free interval, the second 4‐week cycle was applied at a dose of 28 μg/d from day 1 to day 28. Central nervous system prophylaxis was performed for all patients by intrathecal administration of triple agents (methotrexate 12 mg, cytarabine 40 mg, and hydrocortisone 50 mg; 6 times in total). Patients who failed blinatumomab therapy were treated with cytotoxic salvage chemotherapy consisting of high‐dose cytarabine (2 g/m^2^, every 12 hours, days 1‐4), mitoxantrone (12 mg/m^2^, days 1‐4), and etoposide (100 mg/m^2^, days 5‐7). For patients who achieved CR, allo‐HCT was offered as early as possible if a matched sibling donor (MSD) or ≤1 allele mismatched unrelated donor (URD) was available. Donor‐recipient pairs were considered matched when the pair had identical HLA‐A, ‐B, ‐C and ‐DRB1 loci with high‐resolution HLA genotyping. Patients with no available MSD or URD were offered HCT using cord blood (CB) or haploidentical related donor (HID) grafts as an alternative source. For CB transplantation (CBT), minimum HLA typing requirements followed the current practice of low‐resolution typing of HLA‐A and ‐B and high‐resolution typing of HLA‐DRB1. CB units were 4‐6/6 HLA‐A, ‐B, and ‐DRB1 matched to the recipient. To be eligible for myeloablative (MAC), patients had to be younger than 50 years with no signs of organ dysfunction or active infections. The MAC regimen for patients receiving MSD or URD‐HCT consisted of total body irradiation (TBI, 13.2 Gy in total) and cyclophosphamide (120 mg/kg in total). The MAC regimen for patients receiving CBT consisted of TBI (12.0 Gy in total), fludarabine (150 mg/m^2^ in total), and cytarabine (9.0 g/m^2^ in total). Patients of advanced age (≥50 years) or with comorbid conditions or patients receiving HID‐HCT were given an identical reduced‐toxicity conditioning (RTC) regimen consisted of fludarabine (150 mg/m^2^ in total) and busulfan (9.6 mg/kg in total). Graft‐versus‐host disease (GVHD) prophylaxis was attempted by administering calcineurin inhibitors (cyclosporine for MSD transplants and tacrolimus for URD or HID transplants) and methotrexate. Antithymocyte globulin was administered to patients receiving URD or HID grafts. For CBT, tacrolimus and mycophenolate mofetil were used for GVHD prophylaxis. If residual leukemia was detected in the absence of GVHD at 3 months after HCT, immunosuppressants were rapidly discontinued. None were treated with any type of maintenance therapy including blinatumomab after allo‐HCT.

### 2.3 Disease related parameters

The International System for Cytogenetic Nomenclature (ISCN) was used to detect clonal abnormalities,^24^ which were classified into risk subgroups according to the National Comprehensive Cancer Network (NCCN) guidelines. Poor‐risk cytogenetics was defined as complex karyotype defined as 5 or more chromosomal aberrations, hypodiploidy, and *KMT2A* rearrangements, while other abnormalities were classified into standard‐risk cytogenetics. CD19 expression was calculated by proportion of leukemic blasts using flow cytometry (BD Biosciences). CR was defined as ≤5% bone marrow (BM) blasts, absolute neutrophil count >1×10^9^/L, and platelets >100×10^9^/L. Minimal residual disease (MRD) negativity was defined as no detectable blasts using a high‐throughput sequencing method for clonal rearrangements of immunoglobulin gene (assay sensitivity, <10^‐5^), as previously described.^25^ Clonal immunoglobulin rearrangement was assessed by the LymphoTrack® IGH FR1/2/3 assay panel (InVivoScribe Technologies) from a BM sample. Amplified and purified amplicons were measured by Agilent 2100 BioAnalyzer (Agilent Technologies, Inc). For MRD monitoring, the clone of the same sequence with the diagnostic sample was sought after blinatumomab. If any identical sequences to the initial clone were found, the amount of remnant clone was described as the % of total reads.

### 2.4 Statistical analysis

The main end points were CR rate, OS, and cumulative incidence of relapse (CIR). Response rate was compared by Fisher's exact test. Survival curves were plotted using the Kaplan‐Meier method, and subgroups were compared by log‐rank tests. Relapse was calculated using cumulative incidence estimates to accommodate competing death events, and subgroups were compared by Gray test. The prognostic significance of covariates affecting response rate was determined by multiple logistic regression, and covariates affecting OS were determined by Cox proportional hazards regression model. The prognostic significance of covariates affecting CIR was determined using Fine‐Gray proportional hazards regression for competing events. In these models, acute and chronic GVHD were considered time‐dependent covariates. Multivariate analyses were performed using variables with *P*‐value <.10 in prior univariate analyses. All statistical analyses were performed using “R” software version 2.15.1 (R Foundation for Statistical Computing, 2012). Statistical significance was set at *P*‐value <.05.

## 3 RESULTS

### 3.1 Baseline characteristics

Baseline characteristics of 32 patients with R/R Ph‐negative BCP‐ALL treated with blinatumomab are summarized in Table 1. Median leukocyte and platelet counts were 4.7 × 10^9^/L (range, 0.9‐39.3 × 10^9^/L) and 67.5 × 10^9^/L (range, 6.0‐338.0 × 10^9^/L), respectively. No peripheral blood (PB) blasts were observed in 16 (50.0%) patients; 5 patients had ≥50% PB blasts. Median BM blast percentage was 80% (range, 10%‐99%). Five (15.6%) patients had poor‐risk cytogenetics at the time of diagnosis (2 *KMT2A* rearrangements, 2 hypodiploidy, and 1 complex karyotype), and 3 (9.4%) patients showed clonal evolution to complex karyotype at the time of relapse. These 8 patients (25.0%) were placed in a poor‐risk cytogenetics group for the next analysis. Eleven patients (34.4%) were primary refractory to induction chemotherapy, 10 (31.2%) relapsed after consolidation chemotherapy, and 11 (34.4%) relapsed after previous allo‐HCT. Among 21 relapsed patients, median duration of first CR was 12.8 months (range, 1.8‐99.4 months); 11 patients (7 after consolidation chemotherapy, 4 after previous allo‐HCT) had a first CR duration (CRD1) shorter than 12 months. Extramedullary disease (EMD) was observed in 5 (15.6%) patients. Of them, 2 patients (6.2%) had isolated EMD and 3 (9.4%) had EMD with concurrent BM involvement. Median CD19 expression was 88.7% (range, 23.8%‐99.4%).

**Table 1:** Baseline characteristics of patients

Parameters	Value
Age, median (range), y	44 (18‐70)
<50 y, n (%)	23 (71.9)
≥50 y, n (%)	9 (28.1)
Male gender, n (%)	14 (43.7)
Leukocyte count, median (range), ×10^9^/L	4.7 (0.9‐39.3)
<5.0 × 10^9^/L, n (%)	17 (53.1)
≥5.0 × 10^9^/L, n (%)	15 (46.9)
Platelet count, median (range), ×10^9^/L	67.5 (6.0‐338.0)
<50 × 10^9^/L, n (%)	10 (31.2)
≥50 × 10^9^/L, n (%)	22 (68.8)
PB blasts, median (range), %	1 (0‐83)
None	16 (50.0)
1 to <5%, n (%)	4 (12.5)
5 to <50%, n (%)	7 (21.9)
≥50%, n (%)	5 (15.6)
BM blasts, median (range), %	80 (10‐99)
<20%, n (%)	7 (21.9)
20% to <50%, n (%)	5 (15.6)
50% to <75%, n (%)	1 (3.1)
≥75%, n (%)	19 (59.4)
CD19 expression, median (range), %	88.7 (23.8‐99.4)
Cytogenetic risk, n (%)	
Standard‐risk	24 (75.0)
Poor‐risk	8 (25.0)
Prior allo‐HCT, n (%)	11 (34.4)
Disease status, n (%)	
Primary refractory	11 (34.4)
CRD1 <12 mo, n (%)	11 (34.4)
CRD1 ≥12 mo, n (%)	10 (31.2)
CRD1, median (range), months	12.8 (1.8‐99.4)
Disease site, n (%)	
BM alone	27 (84.4)
BM + extramedullary	3 (9.4)
Extramedullary alone	2 (6.2)

Abbreviations: Allo‐HCT, allogeneic hematopoietic cell transplantation; BM, bone marrow; CRD1, first complete remission duration; PB, peripheral blood.

### 3.2 Response to blinatumomab

**Figure 1:**
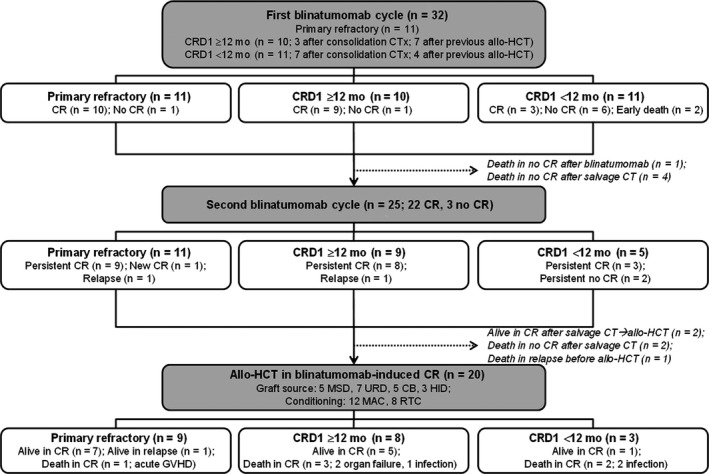
Flowchart of patients included in this study and overall response. CRD1 indicates first complete remission duration; allo‐HCT, allogeneic hematopoietic cell transplantation; CB, cord blood; CR, complete remission; CT, chemotherapy; GVHD, graft‐versus‐host disease; HID, haploidentical related donor; MAC, myeloablative conditioning; MSD, matched sibling donor; RTC, reduced‐toxicity conditioning; URD, unrelated donor

A patient flowchart is provided in Figure 1. After the first blinatumomab cycle, 22 (68.8%) of 32 patients achieved CR with full neutrophil and platelet recovery as defined (10/11 patients with primary refractory disease, 9/10 relapsed patients with CRD1 ≥12 months, 3/11 relapsed patients with CRD1 <12 months). No cases of CR with partial or incomplete hematologic recovery were observed. Two patients (6.2%) who had relapsed within 12 months after previous allo‐HCT died of sepsis and tumor lysis syndrome during the first blinatumomab cycle, and the remaining 8 (25.0%) patients failed to achieve CR. Twenty‐five patients (22 CR, 3 no CR) were available for assessment of their response to the second blinatumomab cycle. Of those, 20 of 22 patients who had CR post‐first cycle remained in persistent CR with full hematologic recovery. In addition, one patient with primary refractory disease who had no CR post‐first cycle achieved new CR with full hematologic recovery at the end of the second cycle. However, 2 patients who achieved CR post‐first cycle relapsed at the end of the second cycle. The remaining 2 patients who had no CR post‐first cycle showed persistent no CR. Among 20 patients with persistent CR, 12 patients were evaluable for MRD assessment. Of those, 9 patients (75.0%) achieved MRD negativity (8 patients at the end of the first cycle and 1 patient at the end of the second cycle; Table 2).

**Table 2:** Response to blinatumomab treatment and allo‐HCT realization

Parameters	Value
At the end of the first cycle (n = 32)	
CR, n (%)	22/32 (68.8)
Primary refractory	10/11
CRD1 ≥12 mo	9/10
CRD1 <12 mo	3/11
No response, n (%)	8/32 (25.0)
Early death, n (%)	2/32 (6.2)
At the end of the second cycle (n = 25)	
Persistent + new CR, n (%)	21/25 (84.0)
Primary refractory	10/11
CRD1 ≥12 mo	8/9
CRD1 <12 mo	3/5
No response, n (%)	2/25 (8.0)
Relapse, n (%)	2/25 (8.0)
MRD negativity in patients with persistent CR, n (%)	9/12 (75.0)
At the end of the first cycle	8 (66.7)
At the end of the second cycle	1 (8.3)
Allo‐HCT in blinatumomab‐induced CR, n (%)	20/32 (62.5)
Graft source, n (%)	
Matched sibling donor	5 (25.0)
Unrelated donor	7 (35.0)
Cord blood	5 (25.0)
Haploidentical related donor	3 (15.0)
Conditioning intensity, n (%)	
Myeloablative conditioning	12 (60.0)
Reduced‐toxicity conditioning	8 (40.0)
Time to allo‐HCT, median (range), days	116 (104‐172)
Cumulative incidence of acute GVHD at 100 d, % (95% CI)	20.7 (6.1‐41.3)
Cumulative incidence of chronic GVHD at 1 y, % (95% CI)	35.0 (13.5‐57.7)
Transplant‐related mortality at 1 y, % (95% CI)	29.3 (3.5‐48.2)
Overall survival rate at 1 y, % (95% CI)	70.7 (42.7‐86.8)

Abbreviations: Allo‐HCT, allogeneic hematopoietic cell transplantation; CI, confidence interval; CR, complete remission; CRD1, first complete remission duration; GVHD, graft‐versus‐host disease; MRD, minimal residual disease.

CR rate was lower in patients with poor disease status (CRD1 <12 months vs CRD1 ≥12 months vs primary refractory; *P* = .004), poor‐risk cytogenetics (poor‐risk vs standard‐risk; *P* = .009), and EMD (positive vs. negative; *P* = .037) at the time of blinatumomab. In multivariate analysis, CRD1 <12 months was an independent predictor for poorer response to blinatumomab (OR, 0.037; 95% CI 0.01‐0.43; *P* = .008) (Table 3).

**Table 3:** Multivariate analysis of factors affecting response to blinatumomab and overall survival

Variables	Response to blinatumomab				Overall survival			
	Univariate		Multivariate		Univariate		Multivariate	
	%	*P*	OR (95% CI)	*P*	% at 1 year	*P*	HR (95% CI)	*P*
Age								
<50 y (n = 23)	69.6	.681			62.0	.141		
≥50 y (n = 9)	55.6				38.9			
Gender								
Male (n = 14)	50.0	.142			49.1	.488		
Female (n = 18)	77.8				60.2			
Leukocyte count								
<5.0 × 10^9^/L (n = 17)	76.5	.266			70.5	.082		
≥5.0 × 10^9^/L (n = 15)	53.3				40.0			
PB blasts								
<5% (n = 20)	75.0	.250			70.0	.015	1	—
≥5% (n = 12)	50.0				33.3		6.75 (1.74‐26.12)	.006
BM blasts								
<50% (n = 12)	66.7	1.000			50.9	.842		
≥50% (n = 20)	65.0				57.8			
CD19 expression								
<95% (n = 18)	72.2	.423			61.1	.139		
≥95% (n = 14)	55.6				40.0			
Cytogenetic risk								
Standard‐risk (n = 24)	79.2	.009			65.9	<.001		
Poor‐risk (n = 8)	25.0				25.0			
Prior allo‐HCT								
No (n = 21)	71.4	.442			61.1	.121		
Yes (n = 11)	54.5				45.5			
Disease status								
Primary refractory (n = 11)	90.9	.004	1	—	85.7	<.001	1	—
CRD1 ≥12 mo (n = 10)	80.0		0.400 (0.03‐5.25)	.485	80.0		1.23 (0.12‐12.02)	.859
CRD1 <12 mo (n = 11)	27.3		0.037 (0.01‐0.43)	.008	9.1		17.93 (3.13‐102.5)	.001
Extramedullary disease								
No (n = 27)	74.1	.037			67.2	<.001	1	—
Yes (n = 5)	20.0				0.0		8.76 (1.69‐45.3)	.009

Abbreviations: allo‐HCT, allogeneic hematopoietic cell transplantation; CI, confidence interval; CRD1, first complete remission duration; HR, hazard ratio; OR, Odds ratio; PB, peripheral blood.

### 3.3 Overall outcomes and prognostic factors

**Figure 2:**
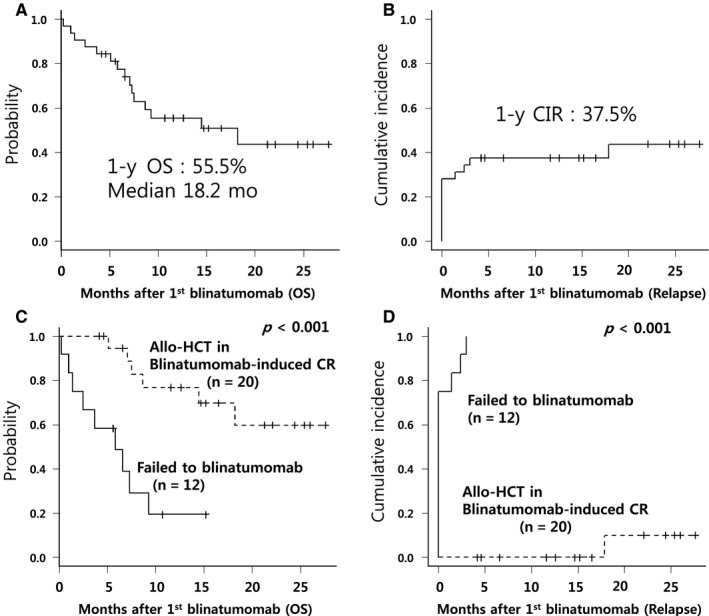
Treatment outcomes of patients treated with blinatumomab with or without allo‐HCT in CR. A, OS for all patients. B, CIR for all patients. C, OS according to application of allo‐HCT in blinatumomab‐induced CR. D, CIR according to application of allo‐HCT in blinatumomab‐induced CR. OS indicates overall survival; Allo‐HCT, allogeneic hematopoietic cell transplantation; CIR, cumulative incidence of relapse; CR, complete remission

To date, 16 (50.0%) of 32 patients remained alive, and 13 of them (40.6%) were in blinatumomab‐induced persistent CR. Sixteen patients died; 8 patients died of progressive leukemia and the remaining 8 patients died of treatment‐related mortality (TRM; 2 during the first blinatumomab cycle, 6 after allo‐HCT). Four patients relapsed at a median CR duration of 2.5 months (range, 1.8‐17.2 months); 2 patients relapsed at the end of the second blinatumomab cycle, 1 patient relapsed while waiting for allo‐HCT, and 1 patient relapsed after allo‐HCT (Figure 1). After a median follow‐up of 15.2 months (range, 4.2‐27.6 months), the 1‐year OS rate for all patients was 55.5% (median OS, 18.2 months), and the 1 year CIR was 37.5% (Figure 2).

**Figure 3:**
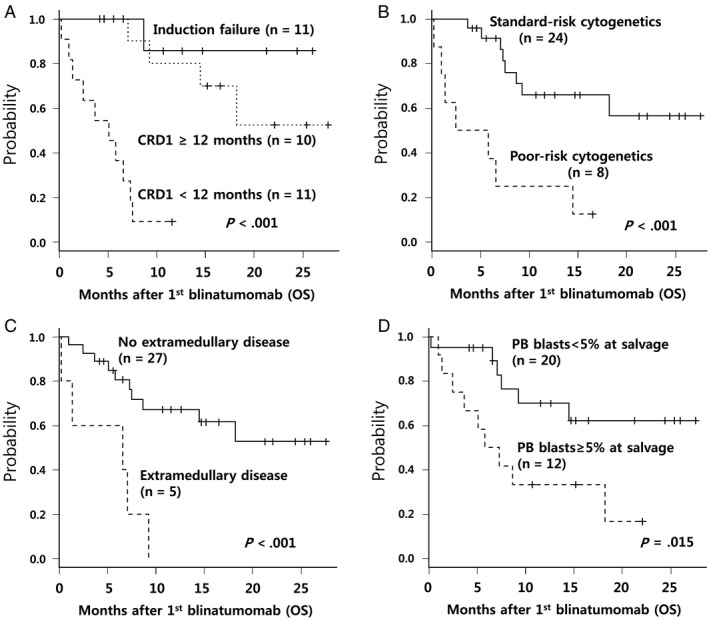
Influence of disease status, cytogenetics, extramedullary disease, and PB blasts on overall survival for all patients treated with blinatumomab. A, Disease status. B, Cytogenetics. C, Extramedullary disease. D, PB blasts. CRD1 indicates first complete remission duration; PB, peripheral blood

Potential factors predicting poorer OS were higher leukocyte count (*P* = .082), disease status with CRD1 <12 months (*P* < .001), poor‐risk cytogenetics (*P* < .001), EMD (*P* < .001), and higher PB blast count (*P* = .015). Multivariate analysis showed that factors independently associated with poorer OS were disease status with CRD1 <12 months (HR, 17.93; 95% CI, 3.13‐102.5; *P* = .001), EMD (HR, 8.76; 95% CI, 1.69‐45.3; *P* = .009), and PB blasts ≥5% (HR, 6.75; 95% CI 1.74‐26.12; *P* = .006) (Table 3; Figure 3).

### 3.4 Outcomes of allo‐HCT following blinatumomab

Twenty (62.5%) of 32 patients proceeded to allo‐HCT in blinatumomab‐induced CR (5 MSD, 7 URD, 5 CB, and 3 HID) at a median time of 116 days (range, 104‐172 days) from the start of blinatumomab. Twelve patients received MAC and 8 patients received RTC. All patients received two cycles of blinatumomab before allo‐HCT. Cumulative incidences of acute GVHD (grades II‐IV) at 100 days and chronic GVHD at 1 year were 20.7% and 35.0%, respectively. Only 1 patient experienced relapse at 17.2 months after allo‐HCT. Six patients died of TRM at a median of 4.0 months (range, 0.9‐14.4 months) after allo‐HCT. The primary causes of TRM were infection (n = 3), organ failure (n = 2), and acute GVHD (n = 1). The 1 year TRM and OS rates were 29.3% and 70.7%, respectively (Table 2; Figure 2).

### 3.5 Blinatumomab‐related toxicity

The most frequent and severe adverse events were hematologic toxicities, such as neutropenia and thrombocytopenia. Neutropenia and thrombocytopenia ≥grade 3 occured in 59.3% and 56.2% of patients, respectively, and febrile neutropenia occurred in 17 (53.1%) patients. Severe CRS or neurologic events were rare in this study. There was only 1 case of grade 2 CRS. Most neurologic events were less than grade 2 and easily manageable (Table 4). Detailed symptoms associated with neurologic events included tremor, dizziness, confusion, ataxia, somnolence, and stroke‐like features.

**Table 4:** Adverse events during the blinatumomab treatment

	Number (%)
Any adverse events	
Neutropenia	27 (84.4)
Anemia	21 (65.6)
Thrombocytopenia	26 (81.2)
Febrile neutropenia	17 (53.1)
Infection	15 (46.9)
Neurologic toxicity	14 (43.7)
Cytokine release syndrome	1 (3.1)
Grade ≥3 adverse events of interest	
Neutropenia	19 (59.3)
Anemia	3 (9.4)
Thrombocytopenia	18 (56.2)
Infection	1 (3.1)
Neurologic toxicity	1 (3.1)
Tumor lysis syndrome	1 (3.1)

## 4 DISCUSSION

Although phase 2 and phase 3 studies of blinatumomab in patients with R/R Ph‐negative BCP‐ALL have been published^17^, ^18^ and several clinical trials in other ALL subgroups have been produced,^19^, ^20^, ^26^ there are few real‐world data of blinatumomab salvage.^27^, ^28^ Our real‐world single center experience of 32 consecutive patients treated with first‐line blinatumomab salvage followed by allo‐HCT is informative in many aspects. Previously, the Korean Adult ALL working party retrospectively collected data of 50 patients with R/R Ph‐negative BCP‐ALL treated with blinatumomab from 16 centers and reported a CR rate of 44.9% and median OS of 7.5 months.^27^ These multicenter data had a short follow‐up duration and a very heterogeneous population of enrolled patients. There were 58% of patients on first‐line salvage, and EMD was observed in 22%. In addition, there was no information of CR duration.

In the current study, 25 (78.1%) patients finished 2 cycles of blinatumomab. Of these, 21 (65.6%) patients achieved persistent CR at 12 weeks, and median survival was 18.2 months after first‐line blinatumomab salvage. All responders achieved true CR with normal neutrophil and platelet counts. The remission rate was higher than 51.0% of the first‐line salvage subgroup in a previous phase 3 study.^21^ In the previous study, 26.0% of patients were refractory to primary therapy and 51.9% of patients were first‐relapse, all of whom had short CR duration less than 12 months. In our data, 11 (34.4%) patients were refractory to primary therapy, and 21 (65.6%) patients were first‐relapse. However, almost half of first‐relapse patients (31.2%) had long CR duration more than 12 months. We found patients with short CR duration showed a poor CR rate of 27.3% to first‐line blinatumomab salvage with poor OS. We suggest that the high overall response to blinatumomab in this study was caused by a higher proportion of patients with long CR duration and patients refractory to primary treatment who showed CR rates of 80.0% and 90.9%, respectively.

Other factors related to poor response to first‐line salvage blinatumomab were also identified and controlled for. These included the effects of high leukocyte or blast counts. Patients were monitored closely during follow‐up and hydroxyurea or cytarabine were quickly initiated with increased leukocyte count or PB blast count. Because of this, only a few patients had high leukocyte or PB blast counts at the time of blinatumomab. In addition, prephase dexamethasone was also administered (80 mg in total) before blinatumomab. As a result, leukocyte and blast counts did not affect responses in this study, in contrast to previous data. Univariate analysis revealed that short CR duration, EMD, and poor‐risk cytogenetics showed a low CR rate. Final multivariate analysis showed that patients with short CR duration only showed poor response to blinatumomab.

Good survival outcome in this study was not only caused by high CR rate but also by a large proportion of successful allo‐HCT with persistent CR after 2 cycles of blinatumomab. Among 21 patients with blinatumomab‐induced CR, 1 patient relapsed while waiting for allo‐HCT. Finally, 20 (62.5%) patients underwent allo‐HCT in blinatumomab‐induced CR. Their 1‐year OS was 70.7% after a median follow‐up duration of 12.9 months. Estimated 1‐year OS was significantly poorer in patients with short CR duration, EMD, poor cytogenetics, and high PB blast count before blinatumomab, similar to the results of blinatumomab response. Multivariate analysis also revealed all parameters except poor‐risk cytogenetics were related to poor OS.

In the current study, poor‐risk cytogenetics were observed in 8 (25.0%) patients, EMD was observed in 5 (15.6%) patients, PB blast count higher than 5% was observed in 12 (37.5%) patients, and BM blast count higher than 50% was observed in 20 (62.5%) patients. In comparison, previous Korean data included the poor‐risk karyotype in 38.1%, EMD in 22.0%, and BM blast count higher than 50% in 81.3% of patients, and which showed 44.9% of overall response.^27^ Thus, the relatively lower proportion of poor‐risk and EMD identified in this study might also contribute to a high rate of successful bridge to allo‐HCT with better outcomes.

For both response and survival, early relapse with short CR duration was a significant factor for poor outcome in this study. Poor outcomes of short CR duration in relapsed patients have been well described in several previous studies,^29^, ^30^ and we showed it again in first‐line blinatumomab salvage. In a previous phase 3 study and subgroup analyses, there were either no patients with first relapse and long CR duration or no comparative data showing poor outcomes associated with short CR duration.^18^, ^21^, ^31^ In another report, CR duration was not significant and EMD was the only significant factor for predicting blinatumomab response.^28^ Regarding EMD in the current study, 3 patients with both BM involvement and EMD all failed to achieve CR, while 1 patient with isolated EMD achieved CR and safely underwent second allo‐HCT.

Although MRD data were available in only 12 complete responders, a high level of MRD response was seen, supporting previous data. Among 65.6% of hematologic CR, MRD negativity was observed in 75.0% of patients. Also, most MRD negative patients safely proceeded to allo‐HCT with very good survival outcomes. A phase 2 trial revealed that the overall response was 43% and the MRD response was 82% among responders.^17^ Another study showed a 36% overall response and 88% MRD response.^19^ Complete and rapid MRD response in earlier salvage lines is very important for better outcomes even after allo‐HCT.^32^ Blinatumomab showed consistently good MRD responses in R/R ALL and also in patients with MRD‐positive remission after primary therapy. These results all show that good survival outcomes were observed in MRD responders.

It is important to consider that some patients will have poor response and post‐blinatumomab relapse, which might suggest resistance to blinatumomab.^33^ For patients with poor responses, combination with other chemoimmunotherapy^34^, ^35^ or urgent allo‐HCT should be considered.

There was only 1 case resembling CRS, but no definite cases of CRS. Neurotoxic events were not frequent. Only 1 patient experienced grade 3 neurotoxicity, and blinatumomab was stopped for 3 days until recovery. Most events were hematologic toxicities and febrile neutropenia, but no one died due to regimen‐related toxicity. To decrease blinatumomab toxicity, unique prephase dexamethasone up to 80 mg was used. There are concerns about the negative effects of dexamethasone, which may inhibit T‐cell activation and proliferation, and the possible effects on the mechanism of blinatumomab.^36^, ^37^, ^38^ However, data have shown that dexamethasone had no impact on blinatumomab induced T‐cell cytotoxicity and proliferation,^39^ and blinatumomab induced T‐cell function does not require interleukin‐2.^40^ Our data support clinical evidence of efficacy and safety of prephase dexamethasone.

In conclusion, we showed that earlier application of blinatumomab for first‐line salvage followed by active allo‐HCT in MRD negative remission significantly improved survival outcomes. However, patients who relapsed with short CR duration remained a challenging problem.

## Data Availability

Original data analyzed in this article are available by first and corresponding author.
